# P-567. Assessing a Community Educational Program’s Impact on Herpes Zoster Vaccine Literacy and Acceptance Among Minoritized Individuals in Vulnerable Communities

**DOI:** 10.1093/ofid/ofaf695.782

**Published:** 2026-01-11

**Authors:** Jacinda Abdul-Mutakabbir, Raheem Abdul-Mutakabbir, Samuel Casey

**Affiliations:** University of California San Diego, La Jolla, CA; University of Michigan School of Public Health, Ann Arbor, Michigan; Congregations Organized for Prophetic Engagement, San Bernardino, California

## Abstract

**Background:**

The acceptance of the herpes zoster vaccine is low among minoritized groups in vulnerable communities despite the higher burden of disease and comorbidities that are known to worsen its presentation. This study describes the effects of a community-based educational intervention to improve vaccine literacy and acceptance among vulnerable individuals from racial and ethnic minority (REM) backgrounds.

**Methods:**

The intervention comprised four interactive, 45-minute educational sessions on the varicella-zoster virus (herpes zoster) and its vaccine. Adults aged 18 years and older were invited to participate in a pre- and post-intervention study that used an anonymous survey to assess their knowledge, attitudes, and behaviors regarding the virus and vaccine. Changes in vaccine literacy were analyzed with the Mann–Whitney U test, while descriptive statistics were used to summarize shifts in vaccine acceptance.

**Results:**

138 participants completed the pre-intervention survey, while 116 (84%) completed the post-intervention survey. All participants identified as a part of a REM group, and 65% were 55 or older. Fifty-four percent reported having at least one comorbid condition, and 48% indicated that they had never received a recommendation to receive the herpes zoster vaccine before they participated in the intervention. Before the intervention, only 57% of the participants believed they were at risk for viral infection; however, 75% said it helped them reassess their risk. We observed significant improvements in vaccine literacy when comparing pre- and post-intervention survey responses, particularly concerning guideline-based recommendations for the herpes zoster vaccine (p< 0.05). Initially, 65% of participants indicated a high likelihood of receiving the herpes zoster vaccine. In contrast, after the intervention, 85% indicated a high likelihood of receiving the herpes zoster vaccine. Furthermore, 93% of post-intervention participants said they would recommend these vaccines to family members or friends after the educational sessions

**Conclusion:**

Community-based educational interventions can greatly enhance vaccine literacy and increase acceptance of the herpes zoster vaccine among vulnerable REM individuals. Further research in this area is needed.

**Disclosures:**

Jacinda Abdul-Mutakabbir, PharmD,MPH, GSK: Advisor/Consultant|GSK: Honoraria|Shionogi Ltd.: Advisor/Consultant|Shionogi Ltd.: Honoraria

